# Isolated Third Cranial Nerve Palsy Following Coronary Angiography: A Rare Neuro-Ophthalmologic Complication

**DOI:** 10.7759/cureus.79244

**Published:** 2025-02-18

**Authors:** Mahmoud M Ismail, Nasser G Al Saedi, Omar S Basamh, Ali H Alsaedi

**Affiliations:** 1 Ophthalmology, King Abdullah Medical City, Makkah, SAU

**Keywords:** cardiac coronary angiography, diplopia, ischemic heart disease, isolated midbrain infarction, isolated third cranial nerve palsy

## Abstract

Neurological complications following cardiac coronary angiography (CAG), such as ischemic stroke and neuro-ophthalmologic syndromes, are uncommon but significant. This report presents a rare case of isolated third cranial nerve palsy that developed after CAG, highlighting the potential risks associated with the procedure. A 51-year-old male with a history of hypertension and ischemic heart disease underwent CAG for non-ST-segment elevation myocardial infarction. Although the procedure and subsequent cardiac imaging results were normal, the patient developed sudden onset binocular diplopia, ptosis, and restricted eye movements in his left eye following CAG, indicative of left third cranial nerve palsy. Initial computed tomography (CT) and computed tomography angiography (CTA) scans were unremarkable, but a magnetic resonance imaging (MRI) revealed multiple recent lacunar infarcts, including one along the path of the left oculomotor nerve, suggesting an embolic cause. Neuro-ophthalmologic complications post-CAG, though rare, require early detection and intervention, emphasizing the need for preventive measures in at-risk patients.

## Introduction

Neurological complications after cardiac catheterization, albeit rare, may include ischemic strokes and neuro-ophthalmologic diseases. The incidence of solitary cranial nerve palsy is exceptionally uncommon [1]. Coronary angiography (CAG) is extensively employed to diagnose and treat coronary artery and valvular heart disorders. Although it is minimally invasive and usually conducted under local anesthesia, it carries a slight risk of complications. Frequent complications include embolic strokes, but less common manifestations, such as cranial nerve involvement, may also arise. Factors that elevate the probability of problems include patient-specific concerns, including advanced age, renal impairment, diabetes, and complex cardiovascular conditions. Elements connected to the procedure, including further interventional measures, can potentially affect outcomes [2]. Although the general risk of substantial complications after cardiac catheterization is minimal, with major incidents happening in less than 2% of cases, the possibility of severe outcomes persists. This article details a rare instance of isolated third cranial nerve palsy that occurred quickly following a CAG operation [3]. This article emphasizes the significance of identifying infrequent yet severe consequences of CAG. This discussion focuses on the clinical appearance, diagnostic findings, and potential processes of isolated oculomotor nerve palsy, highlighting the importance of early discovery and care.

## Case presentation

A 51-year-old man with a history of ischemic heart disease and hypertension presented to the primary hospital with chest pain. He was referred to our hospital for cardiac catheterization after being diagnosed with acute coronary syndrome with non-elevated ST-segment myocardial infarction. The cardiac assessment showed that the left ventricle was normal in size with moderate concentric hypertrophy and normal systolic function (ejection fraction (EF) > 55%). Impaired left ventricular relaxation was evidenced by the transmitral Doppler flow pattern, with no abnormalities in regional wall motion identified. The right ventricle had normal dimensions and functionality. The patient exhibited stability and required no immediate intervention.

**Figure 1:**
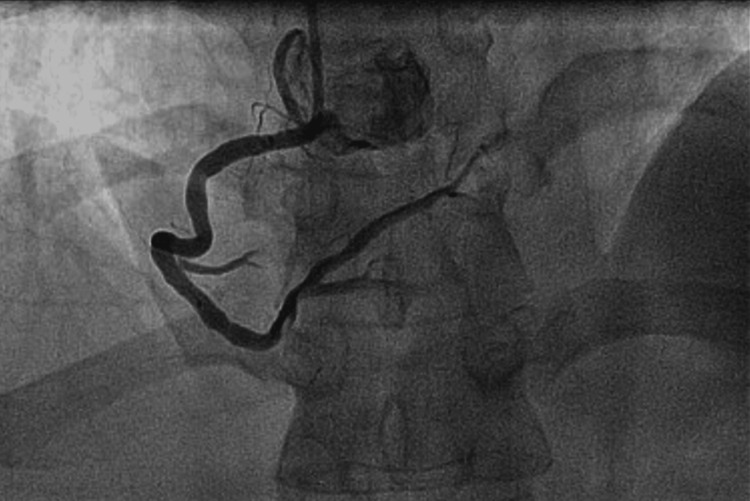
The coronary angiography demonstrating mild non-obstructive coronary artery disease.

As part of dual antiplatelet therapy (DAPT), the patient had been on clopidogrel (75 mg daily) and aspirin (100 mg daily) before the procedure. During the procedure, unfractionated heparin was administered as standard anticoagulation to reduce thrombotic risk. No procedural sedation or additional pre-medications were required beyond standard cardiac catheterization protocols. The CAG was performed uneventfully by a senior interventional cardiologist in a well-equipped setting. There were no procedural complications, prolonged fluoroscopy time, or hemodynamic instability, and the patient remained stable throughout, requiring no immediate intervention (Figure 1).

**Figure 2:**
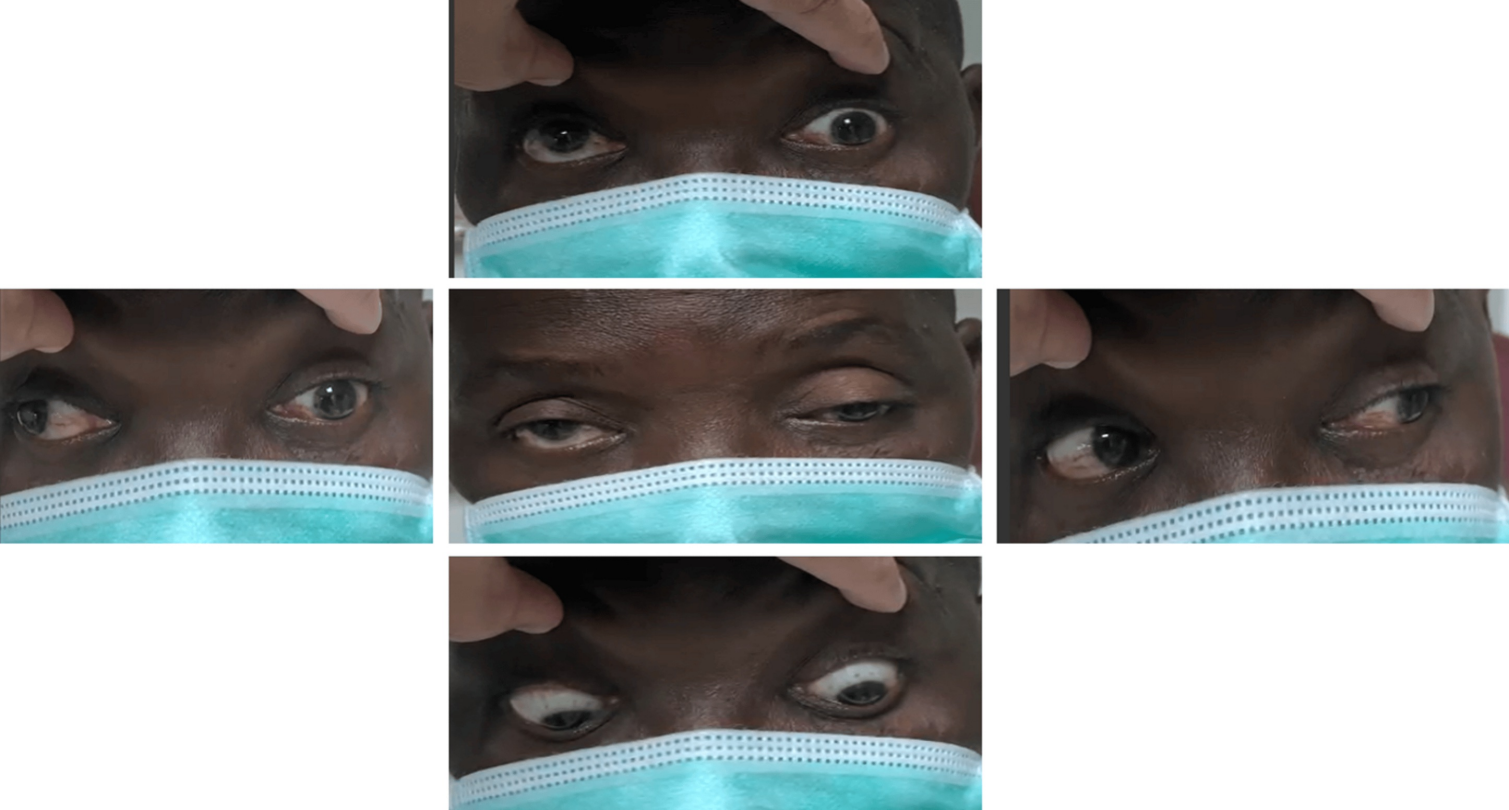
Exotropia on primary gaze with limited adduction, elevation, and depression of the left eye.

Four hours following the procedure, the patient presented with sudden-onset diplopia (double vision), having never previously experienced similar complaints. He did not report any proptosis or pain. Upon examination, the patient exhibited a sluggish pupillary reflex, ptosis of the left eyelid, and impaired adduction, elevation, and depression of the left eye. Additionally, there was left eye exotropia in the primary gaze and diplopia in the down and right gaze, strongly suggestive of a left third nerve palsy with pupillary involvement (Figure 2). The rest of the neurological evaluation was unremarkable.

**Figure 3:**
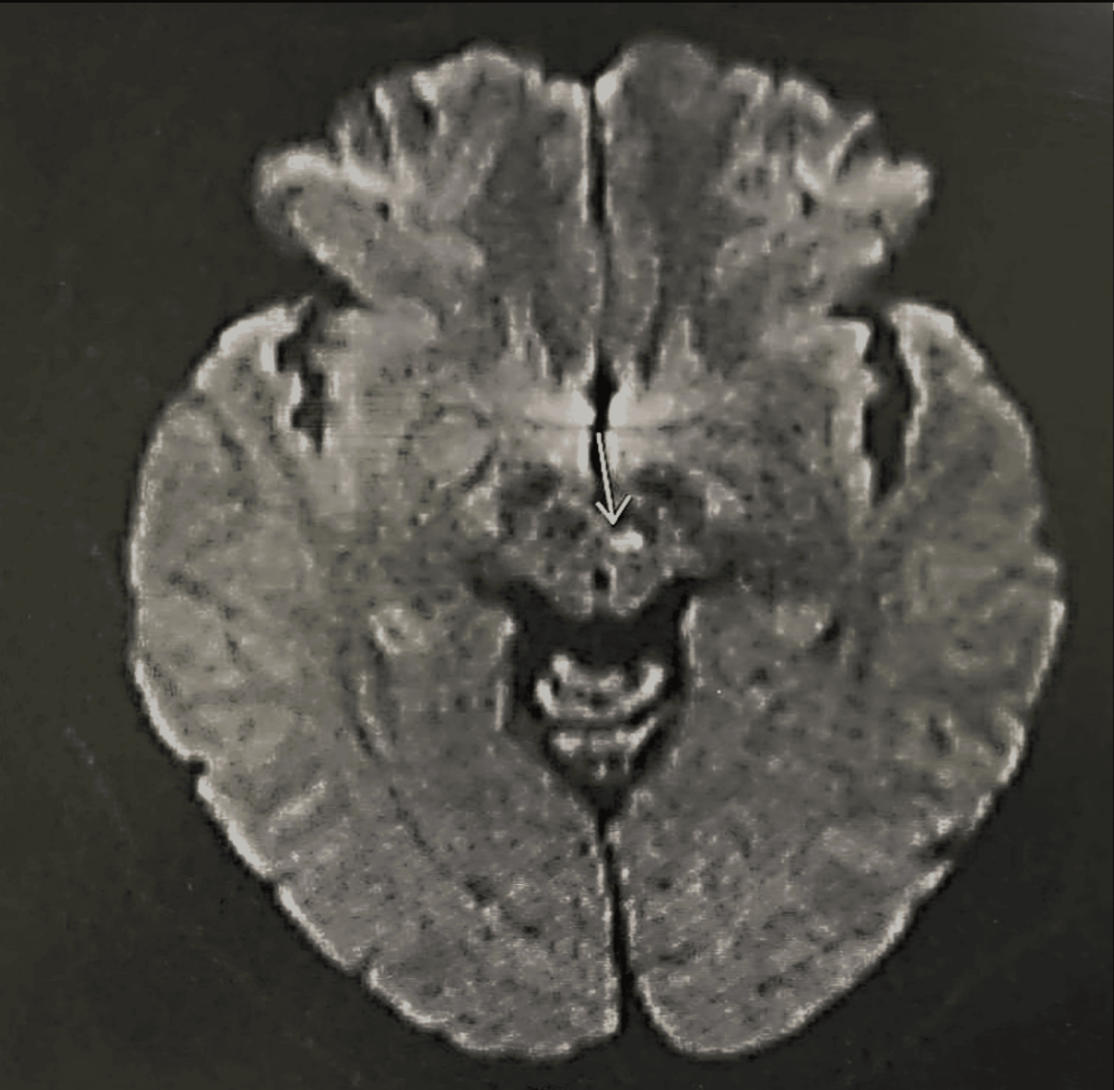
Magnetic resonance imaging shows recent midbrain lacunar infarcts.

An exhaustive laboratory workup was performed, which was normal except for elevated glycated hemoglobin. Given the potential for stroke following cardiac catheterization, emergency head and neck computed tomography (CT) and computed tomography angiography (CTA) were conducted to rule out aneurysm compression or stroke. The results were within normal limits; however, magnetic resonance imaging (MRI) revealed multiple bilateral supra- and infratentorial recent lacunar infarcts. One of these infarcts was embolic and located in the midbrain, following the anticipated path of the left oculomotor nerve (Figure 3).

A comprehensive embolic workup was conducted, but the results were negative. A review of the literature suggests that embolic phenomena can be a potential cause of ischemic strokes following cardiac angiography. Although MRI findings help clarify the underlying mechanism in patients with isolated cranial nerve palsy, the role of small microemboli remains a suggested mechanism, even when CT and magnetic resonance angiography (MRA) neuroimaging are negative. The patient was managed as a stroke case and received standard supportive therapy. Once stabilized, the patient was discharged as he was a pilgrim and desired to return to his home country.

## Discussion

Unilateral isolated oculomotor nerve paralysis after CAG is an uncommon neurological consequence. Various factors, including emboli, anatomical defects, or operative problems, can cause it. Although this particular problem is unusual, similar examples described in the literature provide useful information about its causes and ramifications. Neurological events following CAG are uncommon, with reported rates ranging from 0.03% to 0.6%. Diagnostic techniques have a lower risk (0.03-0.3%) than interventional procedures such as percutaneous coronary interventions (PCIs), which have a risk of 0.3-0.4% [4]. Silent embolic infarctions occur more frequently than symptomatic strokes [5]. Researchers discovered focal diffusion imaging abnormalities in 22% of individuals undergoing retrograde catheterization, despite only 3% having clinical stroke symptoms. The main risk of strokes after CAG is embolic events caused by dislodged thrombus, calcified debris, or cholesterol particles during the operation. Larger catheters and longer operation periods increase the danger. Furthermore, old age, diabetes, hypertension, previous cerebrovascular episodes, and other comorbidities all increase the risk of neurological problems [3]. Anatomical abnormalities, such as the Percheron artery, can potentially exacerbate ischemic stroke symptoms. This unusual vascular variation nourishes both the paramedian thalamus and the rostral midbrain. While its obstruction usually results in bilateral thalamic infarctions, midbrain involvement might cause cranial nerve impairments, such as oculomotor palsy. The rate of artery of Percheron infarction is modest, accounting for about 0.1-2% of all ischemic strokes [6]. Specific cases show variation in clinical presentation. Biller et al. documented a patient with bilateral internuclear ophthalmoplegia, retraction nystagmus, and somnolence as a result of a dorsal tegmental infarction after angioplasty [7]. Liu et al. described two cases of bilateral oculomotor nerve palsy following coronary operations [8]. Another instance reported by Mihaescu et al. involved bilateral ptosis, upgazed paresis, and internuclear ophthalmoplegia caused by a periaqueductal gray matter infarction [9]. These findings highlight the importance of advanced imaging in diagnosis and management. Two hours after the CAG, a patient in one example displayed mild left-sided ptosis, binocular diplopia, and partial left-eye adduction impairment. A brain MRI indicated a midbrain tegmental infarction, underscoring the rarity of unilateral oculomotor nerve palsy following CAG [10]. Other causes of solitary oculomotor nerve palsy are anatomical defects, vasospasm, and immune-mediated responses. For example, a calcified posterior petroclinoid ligament was linked to nerve compression following a minor fall [11]. Furthermore, post-COVID-19 immunization cases with positive ganglioside antibodies point to an immune-mediated mechanism, which may also apply to post-procedural nerve palsies [12,13]. Furthermore, unintentional dural punctures during medical operations have been linked to transitory cranial nerve palsies, comprising the oculomotor nerve as well [14].

## Conclusions

The occurrence of isolated oculomotor nerve palsy due to midbrain infarction post-CAG is exceedingly rare. The interplay of embolic mechanisms, anatomical variations, and patient-specific factors contributes to its complexity. While advanced imaging and current management strategies have improved diagnosis and outcomes, further research is vital to develop standardized treatment approaches for such unique complications.
